# Urothelium proliferation is a trigger for renal crystal deposits in a murine lithogenesis model

**DOI:** 10.1038/s41598-018-34734-8

**Published:** 2018-11-05

**Authors:** Héloïse Bilbault, Joëlle Perez, Léa Huguet, Sophie Vandermeersch, Sandrine Placier, Nahid Tabibzadeh, Vincent Frochot, Emmanuel Letavernier, Dominique Bazin, Michel Daudon, Jean-Philippe Haymann

**Affiliations:** 1 Sorbonne Université, INSERM, UMR_S 1155, AP-HP, Hôpital Tenon, 4 rue de la Chine, 75020 Paris, France; 2 Service d’Explorations Fonctionnelles Multidisciplinaires, Assistance Publique - Hôpitaux de Paris (AP-HP), Hôpital Tenon, 4 rue de la Chine, 75020 Paris, France; 3 Sorbonne Université, GRC n°20, Groupe de Recherche Clinique sur la Lithiase Urinaire, Hôpital Tenon, 4 rue de la Chine, 75020 Paris, France; 4 Lab. Physique des Solides, UMR CNRS 8502, 91405 Orsay, France

## Abstract

Most mouse kidney stone models induce nephrocalcinosis rather than urolithiasis. The aim of our study was to find an accelerated experimental model in order to study the early events of stone formation, that is, at the time of crystal binding to intrarenal urothelium. C57B6 mice exposed to vitamin D supplements and water containing hydroxyl-L-proline, ammonium chloride and calcium chloride were studied for 42 days. A group receiving urothelial cell mitogen Fibroblast Growth Factor 7 (FGF7) was compared to control group receiving saline. Calcium oxalate monohydrate (COM) crystals were detected in urines by day 2 and within urinary spaces in specialized fornix areas in both groups as soon as day 14 with enhanced deposits in FGF7 group compared to controls at day 21. Urothelial cells proliferation, uroplakin III downregulation and de novo expression of osteopontin receptor CD44 detected in FGF7 group, were delayed in the control group (day 42). Crystal aggregates within specialized fornix areas by day 42 were located in urinary spaces but also within and under a multilayered metaplastic urothelium, simultaneous to macrophages influx. Point of note, administration of a normal diet by day 21 was responsible for a spontaneous crystal clearance. Our data show that under supersaturation conditions, urothelial cell proliferation and calcium oxalate crystal retention occur within specialized fornix areas. Enhanced crystal deposits following FGF7 administration suggest that urothelium proliferation would be a relevant trigger for renal stone formation.

## Introduction

Prevalence of urolithiasis is occurring in 8–10% of the general population and is mainly related to environmental factors, such as low water intake altogether with the western diet^1^. Calcium stones are encountered in 80% of cases and contain often a mixture of calcium oxalates and calcium phosphates. Among calcium oxalate crystals, calcium oxalate monohydrate crystalline form (COM) is oxalate dependent, whereas calcium oxalate dihydrate (COD) is calcium dependent^1^. Surprisingly, most rodent models mimic nephrocalcinosis, *i.e*. crystal deposition within renal parenchyma (tubules and interstitium) and only very few within urinary tracts contrary to most human renal stone formers^2,3^. These findings explain why the mechanisms of crystal adhesion have been studied mostly in the tubules^4,5^ where tubular lesions following obstruction appear as a hallmark feature for crystal attachment with a demonstrated key role of CD44 receptor and its ligands osteopontin (OPN) and hyaluronic acid (HA). In these models, tubular proliferation was considered as a key player for crystal clearance process, enabling crystal exclusion from the lumen^6^. Alternatively, urothelial proliferation could be a trigger leading to a cell phenotype change favoring crystal adhesion.

Our study aimed to create a mouse model of renal stone disease in order to study the early events leading to crystal deposition/retention within urinary cavities. In addition to calcium chloride and vitamin D, we administrated hydroxyproline supplements (HLP) in accordance with previous rodent models^3,7^, but also ammonium chloride with the aim of decreasing urinary macromolecular inhibitors. We also tested the potential effect of FGF7 administration on kidney crystal retention, as FGF7 was previously shown to induce proliferation of urothelial cells *in vivo* while changing urothelial phenotype^8,9^.

Therefore, we demonstrate that renal specialized fornices (i.e. forniceal pouches reaching deep into the outer medulla)^10^ are the main location of crystal deposition upon exposure to urine supersaturation and that administration of FGF7 without any previous renal injury enhances renal crystal retention.

## Materials and Methods

### Materials

C56B7 female mice aged 6 weeks (17–18 g) were purchased from Janvier Labs (France). All mice had free access to food and water, and their consumption was recorded daily. The mice were weighed weekly to check their growth. Their urine was collected daily and the calciuria, oxaluria, pH and crystal count were recorded. All animal studies were conducted in accordance with National Institutes of Health (NIH) guidelines for the use and care of laboratory animals and under an active protocol approved by the Institutional Animal Care and Use Committee (National Ethical Study Committee on Animal Experiments, number 05, reference #382,2015032611522460 v3).

### Experimental induction of renal calcium crystal formation

After one week of acclimatization to our animal facilities, the mice under a regular diet were divided into four groups: control, FGF7, HLP diet and HLP + FGF7. Groups HLP and HLP + FGF7 received vitamin D (1000UI) 3 times a week and drank water containing 4% hydroxyl-L-proline, ammonium chloride (0.28 M), calcium chloride (0.25%) and sugar (1%) until sacrifice by days 15, 21 and 42. The aim of this diet was to increase urine supersaturation by increasing urine concentration of both calcium and oxalate but also by trying to decrease urine solubilizing factors such as citrate. FGF7 and HLP + FGF7 groups received an intraperitoneal injection (i.p.) of Fibroblast Growth Factor 7 (FGF7) on days 7 and 8, then once a week until sacrifice to induce urothelial cell proliferation. HLP and control groups received NaCl 0.9% i.p. instead of FGF7. The numbers of mice were: N = 45 in group HLP + FGF7 (10, 25 and 10 sacrificed by days 15, 21 and 42 respectively); N = 40 in group HLP (10, 20, 10 sacrificed by days 15, 21 and 42 respectively); N = 15 in FGF7 and control groups (5 sacrificed by days 15, 21 and 42 for each of both groups). To test the reversibility of the process, normal water was given to groups HLP and HLP + FGF7 from day 21 to day 42 (N = 5/group).

### Detection and quantification of renal calcium crystals in specialized fornices

Kidneys were processed for localization and identification of crystals. 10 μm kidney cuts were performed in a sagittal axis from the convexity to the pedicle as previously described^8^ and then analyzed using a polarizing microscope, an infrared imager (Spotlight 400 FT-IR imaging system from Perkin Elmer) and a Field Emission Scanning Electron Microscope (Zeiss SUPRA55-VP SEM) with no coating. Quantification of CaOx crystals was carried out by examining the sections at magnification ×100. Instead of quantification of the number of crystals per section which was difficult due to the presence of crystal aggregates, we expressed the number of sections (expressed as percentage) with at least one crystal detected (20 sections were analysed in a blinded fashion for each kidney). HLP and HLP + FGF7 groups were compared at different time points.

### Immunohistochemistry and Immunofluorescence studies

primary antibodies anti-Ki67, anti OPN (Abcam, Paris, France), anti-UP III, anti HAS3, anti MGP (Santa Cruz Biotech, Santa Cruz, CA) and anti CD44 (Exbio, Prague, Czech Republic) were used. Peroxidase-labeled polyclonal antibodies anti-rabbit, anti-goat, anti-rat immunoglobulin, all obtained from Histofine Incorporated (Tokyo, Japan) were used as secondary antibodies. BrdU was given in drank water from day 0 (or when indicated) to sacrifice. Kidney sections were fixed in 1:1 acetone. DNA denaturation was induced by hydrochloric acid 1 M. Monoclonal FITC-labeled anti-BrdU antibody (BD Biosciences, San Jose, CA) and fluorescent polyclonal Alexa goat anti-rabbit and anti-rat (Invitrogen, Carlsbad, CA) were used.

### RT PCR analysis

Whole kidney total RNA was extracted using TRIzol solution and methyl trichloride^11^. After reverse transcriptase (MAXIMA First CDAN synthesis kit, Thermo Fischer Scientific Biosciences, Germany) PCR was performed using a LIGHT CYCLER 480 (Roche Diagnostics, Meylan, France), SyBR green (Roche Diagnostics, Meylan, France) and specific primers and probes purchased from Euronfins (Paris, France). The amount of gene products in the test samples was estimated relatively to the respective standard curves. Values for target genes were normalized to glucuronidase beta (GUSB) ones. Results are expressed as a percentage compared to control group when indicated.

### Statistical analysis

The results are expressed as mean or percentage. Comparisons between groups are performed using a non-parametric Mann Whitney test. p values < 0.05 were considered as significant. Statistical analysis was performed using StatView 5.0 (SAS Institute) and Excel 2013.

## Results

### Localization and identification of crystal deposits in kidneys

In mice receiving supplement of Hydroxy-L-proline (HLP), an endogenous precursor of oxalates, water intake and urine production were reduced compared to control animals (2.1 vs 5.5 mL/mice/24 h) but they all remained healthy and gained weight (average weight:17.5 g by day 42). As shown on Table 1, no renal function impairment was detected in the different groups, while calcemia and serum phosphorus values remained normal in HLP supplement groups. Urinary concentrations of calcium and oxalate were similar in HLP and HLP + FGF7 groups between days 15 and 42 but significantly increased compared to baseline (p < 0.001). The urinary pH decrease from 6.9 to 6.5 in treated animals (explained by ammonium chloride intake) was not different between the two HLP groups. Urinary osmolarity strongly increased in HLP and HLP + FGF7 groups, thus favoring crystal onset.

**Table 1:** Plasma and urinary parameters at baseline, in HLP and in HLP + FGF7 groups at days 15,21 and 42.

	Baseline	HLP Day 15	HLP + FGF7 Day 15	HLP Day 21	HLP + FGF7 Day 21	HLP Day 42	HLP + FGF7 Day 42
**Urine**							
Oxalate mmol/L	0.4 + /− 0.3	6.9 + /−0.5*	6.7 + /−2.8*	6.2 + /−2.9*	6.0 + /−1.6*	7.4 + /−0.6*	7.4 + /−0.4*
Calcium mmol/L	1.4 + /−0.4	6.2 + /−1.0*	6.3 + /−1.5*	5.4 + /−1.3*	5.2 + /−0.9*	5.9 + /−1.6*	4.4 + /− 1.0*
osmolarity	1730 + /− 78	3523 + /−454*	2596 + /−382*	2834 + /−883*	2780 + /−235*	2882 + /−28*	3367 + /−836*
urinary pH	6.9 + /−0.1	6.5 + /−0.1*	6.5 + /−0.3*	6.5 + /−0.3*	6.5 + /−0.3*	6.5 + /−0.2*	6.5 + /−0.3*
**Blood**							
Creatinine μmol/L	14.2 + /−1.7	11.8 + /− 2.1	11.4 + /− 1.7	14.8 + /−2.5	13.7 + /− 1.5	13.7 + /−1.6	13.1 + /− 0.4
Magnesium mmol/L	1.0 + /− 0.1	1.1 + /− 0.1	1.0 + /− 0.1	1.1 + /− 0.1	0.9 + /−0.1	1.1 + /− 0.1	1.0 + /− 0.1
Urea mmol/L	6.0 + /−1.0	7.2 + /−1.2*	9.0 + /−1.2*	5.9 + /− 0.6	6.4 + /−0.7	7.4 + /− 1.7	5.7 + /− 0.6
Phosphate mmol/L	2.2 + /−0.7	1.7 + /−0.2	1.8 + /−0.3	2.2 + /− 0.3	2.4 + /−0.4	2.1 + /− 0.3	2.3 + /− 0.3
Calcium mmol/L	2.2 + /−0.2	2.24 + /−0.1	2.35 + /−0.1	2.3 + /− 0.1	2.35 + /− 0.1	2.30 + /− 0.1	2.4 + /−0.1

^*^Indicates a p value < 0.05 compared to baseline.

**Figure 1:**
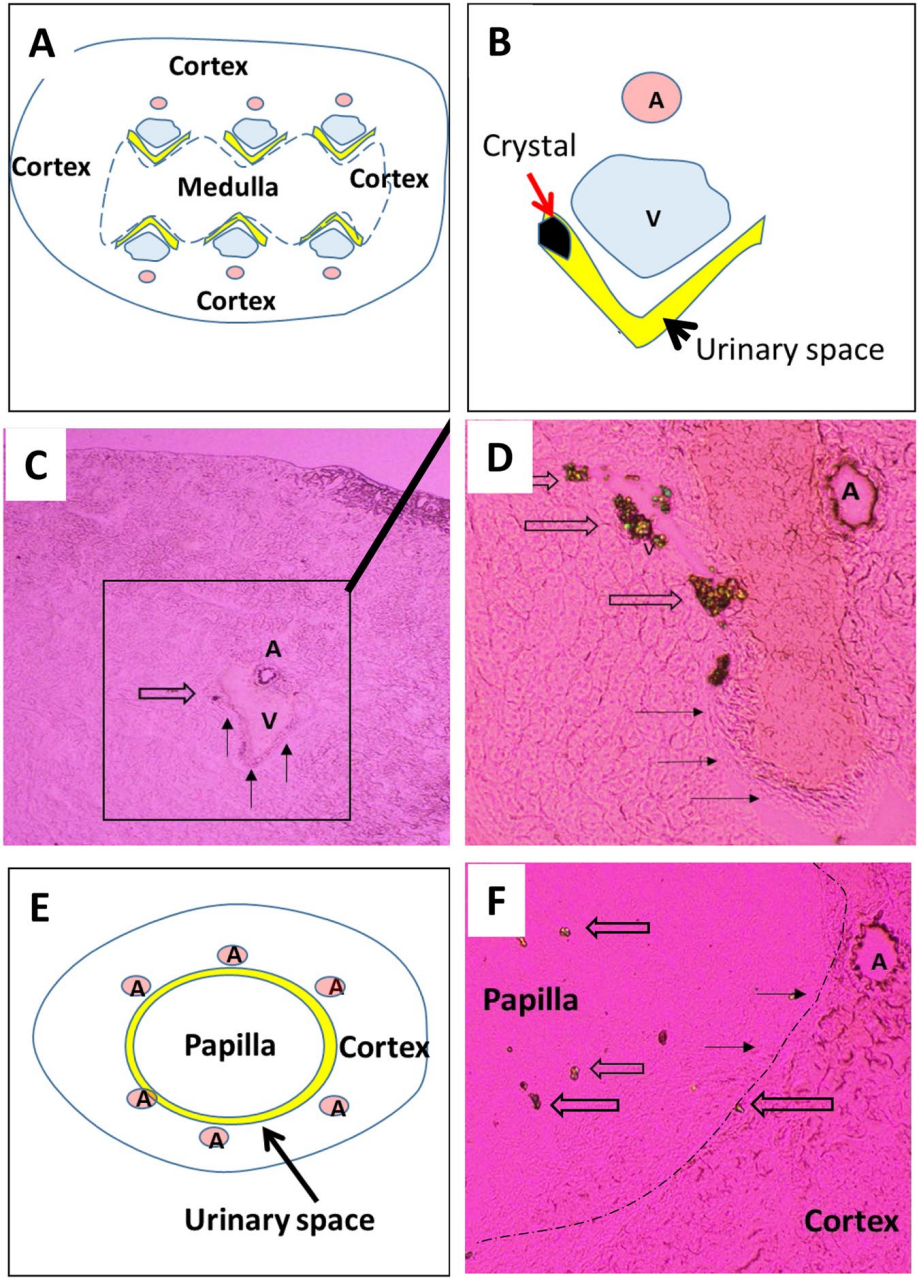
(**A**) Schema representing a kidney sagittal section with urinary spaces corresponding to specialized fornices area per section. (**B**) Magnification of one specialized fornix showing a crystal attached to urothelial cells and close to interlobar arteries and veins (A and V respectively) representing figure (**C**) features. (**C**) Crystal deposit within one renal specialized fornix in the HLP group on days 15 and 42. (**D**) (urinary spaces are indicated by arrows). (**E**) Schema representing a papilla sagittal section and (**F**) Crystals (large arrows) along the urothelium (thin arrow) and within papilla. Frozen sections. Original magnification: (**C**) x40. (**D**,**F**) x100. A = artery. V = vein.

**Figure 2:**
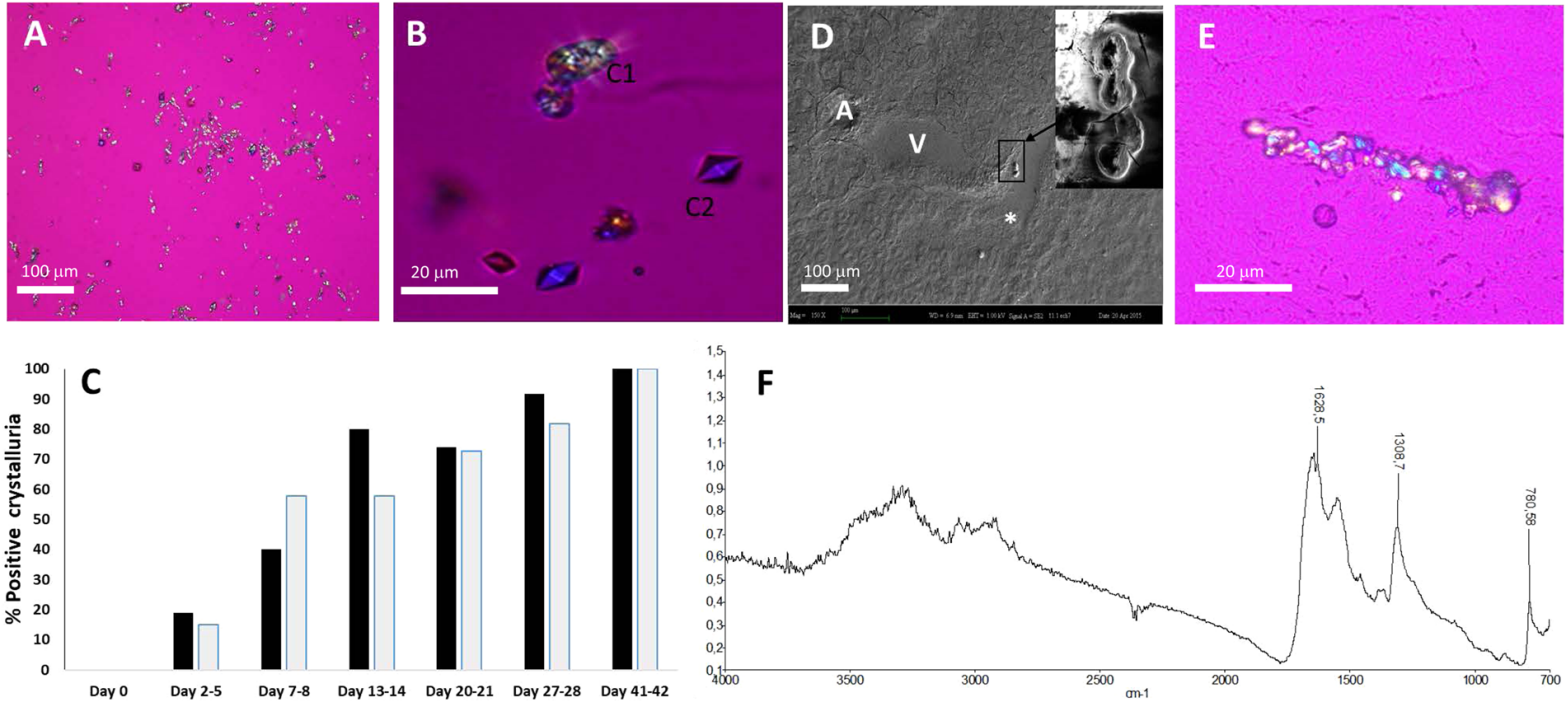
(**A**,**B**) Crystals in urines with COM (C1) and COD (C2) crystals observed at day 15 in HLP group with 2 different magnifications. (**C**) Quantification of positive crystalluria (expressed as a percentage) in HLP and HLP + FGF7 groups during the study (black and grey bars respectively). (**D**) COM crystals in one renal specialized fornix on day 15 detected by SEM analysis. (**E**) COM crystals located in one renal specialized fornix on day 42 detected by polarizing microscope. (**F**) Infrared spectrum of a crystal deposit detected in a specialized fornix, observed by reflection (infrared imager: Spotlight 400 FT-IR imaging system from Perkin Elmer) with a specific COM signature (peaks at 1308 and 780 cm^−1^). Original magnification: (A) X40, (B and E) X400. A = artery. V = vein.

By day 15, crystal deposits were detected within the urinary cavities, near urinary recesses in all kidneys in the HLP group (Fig. 1C). At this location, these urinary cavities form outpocketings called specialized fornices^10^. It is interesting to note that, using a sagittal cutting, up to 6 specialized fornices per section could be identified as shown in our diagram (Fig. 1A), located close to interlobar arteries and veins (Fig. 1B). As shown, from days 15 to 42, most crystals were located in urinary specialized fornices (Fig. 1C,D), and very few within the tubules and in the papillary base (Fig. 1E,F). The use of our specific sagittal cutting enables a follow up of the size and number of crystal deposits with a good reproducibility in this location. As illustrated in Fig. 1C,D, the size and number of crystal deposits were increasing from day 15 to day 42. At day 42, the size reached up to 50 µm in some sections with large crystal aggregates (Fig. 2E). As shown in Fig. 2A,B, many birefringent crystals (under polarizing conditions), very suggestive of COM crystals, were detected in the urines under HLP supplements, with occasionally some non refringent bipyramidal COD (as illustrated in Fig. 2B). Crystalluria was present in more than half of the animals as early as one week after HLP administration (Fig. 2C). It is worthwhile to note that within kidneys, COM was the only crystal species detected as evidenced by their birefringent property (Fig. 2E), dumbbell-shaped morphology (Fig. 2D) and infrared imager specific pattern (Fig. 2F).

### Effect of FGF7 mediated urothelial cell proliferation on crystal retention

**Figure 3:**
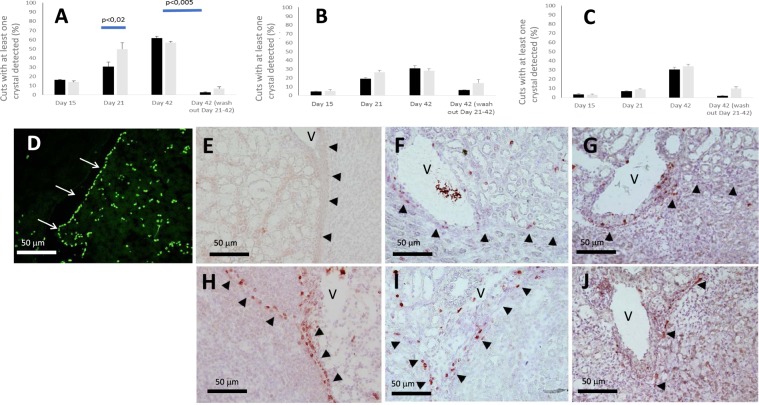
Crystal retention in HLP and HLP + FGF7 kidneys (black and gray bars respectively) during the study course in urinary space (**A**), in tubules (**B**) and in interstitium beneath the urothelium (**C**). Results are expressed in % of sections with the presence of at least one crystal (**D**) BrdU staining after administration of BrdU in drinking water for 15 days. White arrows indicate urinary spaces which are collapsed in frozen tissue sections. KI67 positive staining in HLP and HLP + FGF7 kidneys on day 15 (**E** and **H** respectively), day 21 (**F** and **I** respectively) and day 42 (**G** and **J** respectively). Black arrows indicate urothelial cells surrounding urinary spaces which are collapsed in frozen tissue sections. V = vein.

As shown in Fig. 3A, the number of crystal deposits within specialized fornices increased from days 0 to 42 both in HLP and HLP + FGF7 groups, with a greater amount of crystals in the latter group at day 21 (p < 0.02), though crystalluria was similar between the two groups (Fig. 2C). As shown, crystal deposits were indeed a frequent finding with at least one crystal detected in 60% of sections at day 42 (20 sections were analysed for each kidney). Although very few crystals were detected within tubules and interstitial tissues beneath the urothelium by day 15, their number was increasing significantly by days 21 and 42 (Fig. 3B and C) with no difference between the two groups. Interestingly, after withdrawal of HLP at day 21, renal crystal retention dramatically decreased at day 42 (p < 0.005). As expected, HLP + FGF7 group (receiving FGF7 i.p. at days 14 and 20), displayed an impressive urothelial proliferation assessed by BrdU or KI67 staining at day 15 (Fig. 3D,H respectively), and to a less degree at days 21 and 42 (Fig. 3I,J). Conversely, renal urothelial proliferation was not detected in HLP group at day 15 (Fig. 3E) or in control animals (data not shown) but increased on scarce cells by days 21 and 42 (Fig. 3F,G).

### Effects of FGF7 on urothelial phenotype

**Figure 4:**
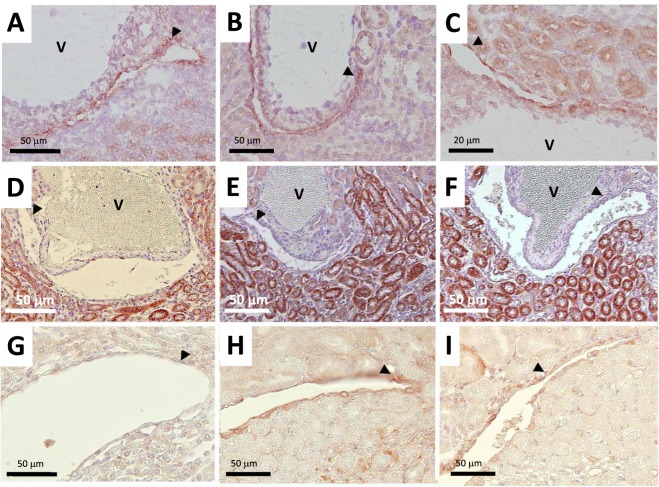
Localization of macromolecule inhibitors osteopontin OPN (**A**–**C**), hyaluronic acid HA (**D**–**F**) and Matrix Gla protein MGP (**G**–**I**) within kidneys on day 21. (**A**,**D** and **G**): control group. (**B**,**E** and **H**): HLP group. C, F and J: HLP + FGF7 group. Black arrows indicate urothelial cells surrounding urinary spaces which are collapsed in frozen tissue sections (**A**–**C**) and open in paraffin-embedded (**D**–**I**) kidney sections. A = artery. V = vein.

**Figure 5:**
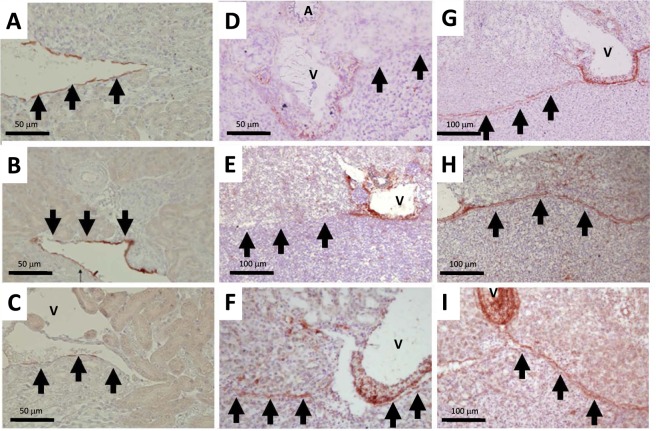
Urothelial phenotype modification during the study. (**A**–**C**)**:** Expression of UPIII positive cells in control group (**A**), HLP group on day 21 (**B**), and HLP + FGF7 group on day 21 (**C**). (**D**–**I**): Expression of CD44 positive cells in control group (**D**), HLP group on day 21 (**E**) and 42 (**F**), 2 days after FGF7 administration (G), HLP + FGF7 group at days 21 (**H**) and 42 (**I**). Paraffin embedded (**A**–**C**) and frozen (**D**–**I**) sections. Positive CD44 cells in the interstitium near interlobar veins (V) are macrophages. Black arrows indicate urothelial cells surrounding urinary spaces which are collapsed in frozen tissue sections (**A**–**C**) and open in paraffin-embedded (**D**–**I**) kidney sections. A = artery. V = vein.

**Figure 6:**
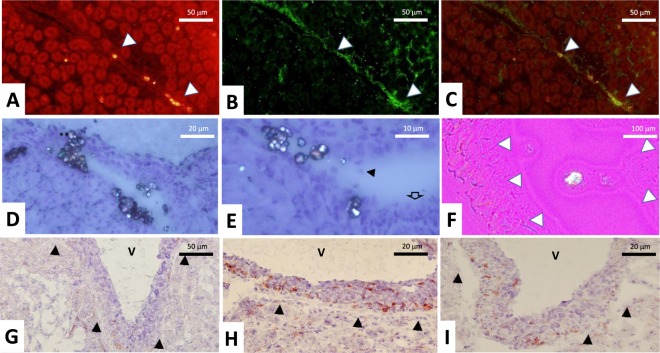
(**A**–**C**) Localization of KI67/CD44 at day 42. KI67 staining (**A**), CD44 staining (**B**) and KI67/CD44 double staining (**C**). (**D**–**F**) Polarized and non-polarized photographs of COM crystals, located in specialized fornix areas at day 42 with some detached urothelial cells in the urinary space (black arrow). Urothelium appears as multilayered (empty arrow). *Crystals* are detected in the urinary space within the multilayered urothelium (**D**,**E**) and covered by the urothelium barrier (**F**). (**G**–**I**) At day 42, F4/80 staining shows fewer positive cells in control group (**G**) compared to HLP (**H**), and HLP + FGF7 group (**I**). White arrows indicate urothelial cells surrounding urinary spaces which are collapsed in most frozen tissue sections.

Administration of FGF7 had no effect either on crystalluria at all time points (Fig. 2C) or renal synthesis of macromolecular inhibitors mRNA such as OPN, HA or Matrix Gla Protein (MGP) at day 21 (data not shown). Of note, OPN was the only molecule, expressed in renal urothelium in physiological conditions, whereas OPN and HA were detected in most inner-medullary tubules and MGP only in vessel walls (Fig. 4A,D,G respectively). Upon HLP supplements with or without FGF7, MGP expression was upregulated on urothelial apical cell membranes at day 21 with no detected modulation for OPN and HA (Fig. 4B,C,E,F,H,I). Of note, following FGF7 administration with HLP supplements, constitutive urothelial UPIII staining disappeared almost completely (Fig. 5A,C), whereas HLP supplements alone did not affect UPIII staining (Fig. 5B). Conversely, FGF7 administration upregulated CD44 within urothelial cells: a very strong staining was detected 24 hours after FGF7 administration alone (Fig. 5G) and at days 21 and 42 (Fig. 5H,I), whereas there was no staining in urothelial cells in control or HLP animals at day 21 (Fig. 5D,E). Of note, a positive urothelial CD44 staining was detected at day 42 in HLP group but located only in some focal areas (Fig. 5F). By day 42, the order of magnitude of proliferating urothelial cells (KI67 positive) was the same in HLP and HLP + FGF7 groups, with most positive KI67 cells also CD44 labeled (Fig. 6A–C). At this time point, in several specialized fornices, urothelium became multilayered in both groups. We noted some crystal deposits trapped within urothelial multilayer, but also urothelial cells detachment creating cell bridges excluding crystal aggregates from the urinary lumen (Fig. 6D–F). In some cases, crystals were detected within interstitium between urinary spaces and interlobar veins (Fig. 6D). In this area, the number of macrophages (F4/80 positive cells) was notably increased in HLP group at day 42 with respect to control animals, especially close to the urothelial barrier (Fig. 6H,G respectively) with no difference detected between HLP and HLP + FGF7 groups (Fig. 6H,I).

## Discussion

Our data show that adding HLP in drinking water altogether with calcium chloride, ammonium chloride and vitamin D induces intra renal calcium deposits in specialized fornices within less than 2 weeks, whereas calcium and vitamin D supplements alone do not induce crystal retention (data not shown). Indeed, after only a few days of HLP supplements, numerous COM crystals are found in most urine samples with the presence of COD crystals occasionally reflecting both urine supersaturation with oxalate and calcium whereas crystalluria rarely occurred after calcium and vitamin D supplements alone, in accordance with previous studies^12^. Intrarenal crystal retention occurred by day 15 at the contact of urothelial cells in the specialized fornices and in few tubules in accordance with previous reports in rats showing that HLP added in drinking water was responsible mostly for crystal aggregates in the renal specialized fornices and pelvis^3,13,14^. However, mice are very resistant to nephrolithiasis, and our model is the first to reproduce a renal stone disease rather than nephrocalcinosis. Indeed, contrary to knockout mouse models which induce nephrocalcinosis^15–17^, our model does not induce kidney failure, hypercalcemia, or failure to thrive and stones are mostly located within urinary cavities. As a matter of fact it was suggested that in mice, both hypercalciuria and hyperoxaluria are required to produce CaP and/or CaOx crystal deposits in kidneys but additional factors also appear to be at play including a remarkable female gender resistance^7^. Here, identification of crystals confirmed the presence of COM only (a well-known oxalo-dependent species) as demonstrated by morphological and infrared analysis, detected as early as day 15 with growth and aggregation processes occurring up to day 42. The decrease of water intake and the increase of urinary osmolarity in HLP groups (probably explained by the unpleasant taste of ammonium chloride which decreases the urine crystal inhibitor citrate through an increased daily acid load^18^) favor the presence of crystals in urines with remarkably high values of urine oxalate, far above measured values in urines of genetic primary hyperoxaluria^19^. The lack of nephrocalcinosis, despite the magnitude of urine oxalate and a hypercalciuria within a 6 to 10 mmol/L range, is an impressive feature suggesting that urinary crystal inhibitors are obviously very efficient in mice. Though we didn’t measure urinary low molecular-weight inhibitors concentrations such as magnesium, pyrophosphate and citrate (known to inhibit crystal nucleation, growth and aggregation^20^), macromolecular inhibitors synthesis such as bikunin, nephrocalcin, OPN, MGP, or HA are not significantly upregulated in whole kidney extracts of HLP treated animals (data not shown). In our model, contrary to genetic hypercalciuric rats where OPN up-regulation is reported^13^, OPN is highly and constitutively expressed noteworthy in urothelial cells and might thus play a key role against crystal nucleation and growth^21^. We show here also an unreported urothelial MGP de novo expression from day 15 upon HLP exposure whereas MGP is (as expected) physiologically expressed within vessels^22^. Altogether, our data suggest a local defensive urothelial mechanism involving OPN and MGP macromolecules acting against crystal retention with a possible upregulation of MGP by hyperoxaluria or COM crystals as demonstrated *in vitro* in tubular cells^12^.

As previously reported and illustrated in Fig. 1, urinary space expansions end at the cortico -medullar junction in the vicinity of interlobar vessels^9^ with six specialized fornices detected in mice (for only one papilla), when performing a kidney sagittal section. We assume that, due to this peculiar anatomy, urine flow is low in the recesses and thus may account for crystal growth and aggregation in the setting of supersaturated urines. All these events may indeed enhance a non-specific local crystal adhesion and account for no crystal retention difference between the two groups at day 15.

However, our data suggest that crystal retention would be enhanced by *in situ* urothelial cell proliferation leading to cell phenotype changes. Indeed, urothelium proliferation was reported to occur early within specialized fornices following renal ischemic injuries, ureteral obstruction or stimulation by pharmacologic agents such as FGF7 via activation of urothelial FGFR2IIIb membrane receptors^8,9^, resulting into the loss of apical UPIII plaques, increased membrane permeability, luminal membrane roughness, urothelial cells multilayer and urothelial desquamation. Accordingly, our data show that FGF7 injections are responsible for an increased retention of crystals in specialized fornices compared to HLP group alone at day 21, altogether with loss of urothelial apical UPIII plaques. However, upregulation of CD44 in proliferating urothelial cells further suggests that a specific attachment of crystals coated with OPN (and/or HA molecules) may be at play. Indeed, crystal retention through the molecular CD44/OPN/crystals complexes is very likely to happen, as previously demonstrated on tubular cells both *in vitro*^5,23^ and *in vivo*^5,6^. Obviously, other OPN urothelial apical membrane receptors such as type αv (β1, β3, β5), α4β1, integrin α9β1^24^ or phosphatidylserine may also be at play and would deserve a specific study. Point of note, at day 42, a delayed crystal mediated - FGF7 independent - urothelial proliferation detected in HLP group, with de novo induction of CD44 receptors (colocalized in KI67 positive cells), could also account for crystal retention. Accordingly, a similar crystal mediated cell proliferation was reported in rat tubular cells following ethylene glycol administration^4^.

The striking finding of renal crystal clearance at day 42 when HLP was removed from drinking water at day 21 is in accordance with a previous study showing crystal removal from kidneys after two weeks despite daily intrabdominal injections of glyoxylate^19^. It suggests that the remarkable ability of mice to clear crystals could be due to various additional processes such as (1) crystal detachment from urothelial cells (2) crystal coating by proliferating urothelial cells excluding crystal from urinary space (illustrated in Fig. 6D–F) (3) powerful macrophage mediated clearance mechanisms^25^. Indeed, crystal translocation from the lumen to the interstitium was reported as a first step of crystal clearance. Then, crystal division into crystallites occurs within interstitium, and finally crystal clearance is achieved by macrophages after endocytosis and lysosome digestion^26^. Though our data do not provide evidence of an increased macrophage activity, the number of macrophages near the urothelium does increase from day 15 after FGF7 injection or HLP supplement, suggesting that modification of urothelium barrier could be a powerful trigger for macrophage migration out of lymphatic vessels located in the vicinities. These speculations, however, require confirmatory data and in particular, in order to assess the key role of OPN, a macromolecule synthesized by urothelial cells that exerts also known chemoattractant and proinflammatory properties on CD44 positive macrophages^26^. Our murine lithogenesis model applied in specific knockout animals could thus be a useful tool to assess the relevance of macromolecule candidates and urothelium receptors in renal crystal retention but also inflammatory response and crystal clearance.

Limitation of the study: Crystal retention to proliferating urothelial cells in specialized fornices could also be favored by a loss of normal pyeloureteric peristalsis mediated by FGF7 administration or the special diet acting on pacemaker cells^27^. This interesting issue is at present unraveled and deserves to be addressed in the future. However, we do not favor this view as no hypotonic or dilated urinary cavities were noticed in kidney sections.

To conclude, urothelial proliferation without any previous injuries appears as a relevant additional stone risk factor in addition to urine supersaturation. Our data suggest that identification of urothelial cells mitogens in our environment such as tobacco^28^, but also in food and drinks could be of clinical relevance and as such would require an epidemiological survey.

### Use of experimental animals

All animal studies were conducted in accordance with National Institutes of Health (NIH) guidelines for the use and care of laboratory animals and under an active protocol approved by the Institutional Animal Care and Use Committee (National Ethical Study Committee on Animal Experiments, number 05, reference #382,2015032611522460 v3).
